# Synergistic Induction of Apoptosis by Boswellic Acid and Cisplatin in A549 Lung Cancer Cells Through NF-κB Modulation and p53 Pathway Activation

**DOI:** 10.3390/cimb47090785

**Published:** 2025-09-22

**Authors:** Mehmet Uğur Karabat, Mehmet Cudi Tuncer

**Affiliations:** 1 Department of Histology and Embryology, Faculty of Medicine, Dicle University, Diyarbakır 21090, Turkey; ugurkrbt@hotmail.com; 2 Department of Anatomy, Faculty of Medicine, Dicle University, Diyarbakır 21090, Turkey

**Keywords:** boswellic acid, lung cancer, apoptosis, cell death, NF-κB, AKBA, cisplatin resistance

## Abstract

The increasing resistance to chemotherapeutic agents in lung cancer significantly contributes to its high mortality. Natural compounds such as acetyl-11-keto-β-boswellic acid (AKBA) have emerged as promising adjuncts to standard therapies. This study investigated the synergistic apoptotic and cytotoxic effects of AKBA in combination with cisplatin (Cis) on A549 non-small-cell lung cancer (NSCLC) cells. Cell viability, apoptosis, and gene expression were evaluated using MTS assay, Annexin V-FITC/PI staining, caspase activity, RT-qPCR, and ELISA, complemented by molecular docking (AKBA–p53) and molecular dynamics (AKBA–p53 and Cis–p53) analyses. Combined AKBA + Cis treatment significantly enhanced apoptosis and reduced cell viability compared to monotherapies (*p* < 0.001), accompanied by upregulation of p53 and caspase-3 and suppression of NF-κB. In silico results further supported direct and stable binding of p53, particularly with AKBA. These findings indicate that AKBA synergizes with Cis to potentiate apoptotic and anti-inflammatory responses in NSCLC and may provide a novel dose-sparing strategy with improved therapeutic efficacy, warranting further in vivo validation.

## 1. Introduction

Lung cancer remains one of the leading causes of cancer-related mortality worldwide, accounting for approximately 22% of all cancer deaths, with NSCLC comprising nearly 85% of cases [1,2]. Despite progress in diagnostic and therapeutic approaches, the overall prognosis of NSCLC remains poor, largely due to the development of resistance to conventional chemotherapeutic regimens. Cis, a platinum-based drug, is widely employed in the management of NSCLC, but its clinical efficacy is frequently limited by intrinsic and acquired resistance as well as severe side effects, including nephrotoxicity and neurotoxicity [3]. Multiple mechanisms contribute to Cis resistance, such as enhanced DNA repair capacity, inhibition of apoptosis, overexpression of drug efflux pumps, and activation of autophagy, which collectively diminish drug effectiveness and highlight the urgent need for adjunctive treatment strategies targeting these pathways [4,5].

In this context, natural compounds have gained considerable attention as potential therapeutic adjuncts due to their pleiotropic biological effects and relatively favorable toxicity profiles. Natural products not only possess inherent anti-cancer activity but can also act synergistically with established chemotherapeutic drugs to enhance efficacy while minimizing systemic toxicity [6]. Among these, acetyl-11-keto-β-boswellic acid (AKBA), a pentacyclic triterpenoid isolated from the resin of Boswellia serrata, has emerged as a promising candidate. AKBA has demonstrated anti-inflammatory, antioxidant, and anti-cancer effects through multiple mechanisms, including the suppression of NF-κB activation, inhibition of IKK signaling, and induction of apoptosis [7,8,9]. These findings suggest that AKBA may overcome key pathways associated with chemotherapy resistance.

Importantly, the therapeutic potential of AKBA in combination with conventional drugs has already been partially explored. Previous studies have shown that co-treatment with AKBA and Cis in NSCLC models promotes G0/G1 phase arrest, induces apoptosis, and suppresses autophagy through p21-dependent signaling [10]. These results indicate that AKBA not only potentiates the cytotoxic efficacy of Cis but may also counteract resistance mechanisms such as autophagy activation. Such evidence provides a compelling rationale for further exploring the AKBA–Cis combination in NSCLC.

Nevertheless, despite these promising findings, the molecular mechanisms underlying the synergistic action of AKBA and Cis remain insufficiently characterized. In particular, it is unclear how this combination regulates key apoptotic genes such as p53 and caspase-3, or how it modulates inflammation-associated transcription factors like NF-κB. Furthermore, structural insights into the direct interaction between AKBA and apoptosis regulators are lacking, which limits our understanding of its mechanistic basis.

In light of these gaps, the present study aimed to investigate the combined effects of AKBA and Cis on cytotoxicity and apoptosis in A549 NSCLC cells. We focused on the transcriptional regulation of p53, caspase-3, and NF-κB, complemented by molecular docking and molecular dynamics simulations to elucidate the potential binding of AKBA to p53. Our hypothesis is that AKBA synergizes with Cis to enhance apoptotic signaling and suppress inflammatory pathways, thereby providing a dose-sparing strategy with reduced toxicity and improved therapeutic efficacy in NSCLC.

## 2. Material and Methods

### 2.1. Maintenance and Preparation of A549 NSCLC Cells for In Vitro Assays

The human NSCLC cell line A549 (ATCC^®^ CCL-185™) was obtained from the American Type Culture Collection (ATCC, Manassas, VA, USA). Cells were maintained in RPMI-1640 medium (Gibco, Thermo Fisher Scientific, Waltham, MA, USA) supplemented with 10% heat-inactivated fetal bovine serum (FBS; Gibco, Thermo Fisher Scientific, Waltham, MA, USA) and 1% penicillin–streptomycin (100 U/mL penicillin and 100 µg/mL streptomycin; Gibco, Thermo Fisher Scientific, Waltham, MA, USA). Cultures were incubated at 37 °C in a humidified atmosphere containing 5% CO_2_. Cells were subcultured at 70–80% confluence using 0.25% trypsin-EDTA (Gibco) and seeded at appropriate densities for subsequent assays. All experiments were performed using cells at passages between 3 and 10 to ensure reproducibility and maintain phenotypic consistency. To ensure culture integrity, cells were routinely monitored for morphology and tested negative for mycoplasma contamination.

### 2.2. Evaluation of A549 Cell Viability Following AKBA Monotherapy

Acetyl-11-keto-β-boswellic acid (AKBA; ≥98% purity, CAS No. 67416-61-9) was purchased from Santa Cruz Biotechnology (Dallas, TX, USA). The effect of AKBA on A549 cell viability was assessed using the MTS assay (Abcam, Cambridge, Cambridgeshire, United Kingdom; ab197010). A549 cells were seeded at a density of 5 × 10^3^ cells per well in 100 µL of complete medium in 96-well flat-bottom plates and allowed to adhere overnight in complete medium. The following day, cells were treated with various concentrations of AKBA (0, 10, 25, 50, 100, and 200 µM) in a final volume of 100 µL/well and incubated for 48 h under standard culture conditions (37 °C, 5% CO_2_).

After the incubation period, 20 µL of MTS reagent was added to each well, and plates were further incubated for 2 h at 37 °C. Absorbance was measured at 490 nm using a microplate reader (BioTek Synergy HTX, Agilent Technologies, Santa Clara, CA, USA). Cell viability was expressed as a percentage relative to untreated control cells. All experiments were performed in triplicate, and each condition was tested in at least three independent biological replicates.

### 2.3. Assessment of Cytotoxicity in A549 Cells Treated with Combined AKBA and Cis

To evaluate the combinatory cytotoxic effects of AKBA and Cis on A549 cells, dose-optimization experiments were performed using AKBA (acetyl-11-keto-β-boswellic acid, ≥98% purity, purchased from Santa Cruz Biotechnology, Dallas, TX, USA) at 0, 10, 25, 50, 100, and 200 µM, and Cis (cisplatin, ≥ 99% purity, obtained from Thermo Fisher Scientific, Waltham, MA, USA) at 0, 1, 2, 3, 4, and 5 µM. A549 cells were seeded in 96-well flat-bottom plates at a density of 5 × 10^3^ cells/well in 100 µL of complete medium and allowed to adhere overnight. The following day, cells were treated with the respective concentrations of AKBA and Cis, either alone or in combination, in a final volume of 100 µL/well.

Following treatment, cell viability was assessed using the MTS assay (Abcam, UK). After 48 h of incubation, 20 µL of MTS reagent was added directly to each well, and plates were incubated for an additional 2 h at 37 °C. Absorbance was measured at 490 nm using a BioTek Synergy HTX microplate reader. Viability percentages were calculated relative to untreated controls. Each condition was tested in triplicate in three independent experiments.

All combinations of AKBA (10–200 µM) with Cis (1–5 µM) were evaluated in triplicate using the MTS assay, and the raw viability data are provided Table S1. Among these, the combination of AKBA 20 µM + Cis 1 µM was selected for detailed presentation in the main text because it achieved approximately 50% inhibition of cell viability, corresponding to the IC_50_ point, and yielded the most representative synergistic outcome (CI = 0.894).

### 2.4. Quantification of Caspase-3/7 Activity in AKBA- and Cis-Treated A549 Cells

To evaluate apoptosis-related enzymatic activity, the Caspase-Glo^®^ 3/7 Assay kit (Promega, Madison, WI, USA) was used according to the manufacturer’s protocol. A549 cells were seeded in 96-well white-walled plates at a density of 5 × 10^3^ cells/well in 100 µL of complete medium and allowed to attach overnight. After experimental treatments, cells were equilibrated to room temperature for 30 min. An equal volume (100 µL) of Caspase-Glo^®^ 3/7 reagent was added to each well, and the plate was gently mixed for 30 s on an orbital shaker.

The samples were then incubated in the dark at room temperature for 1 h. Luminescence was measured using a compatible microplate luminometer (e.g., BioTek Synergy HTX). Caspase-3/7 activity was quantified by comparing relative light units (RLUs) from treated groups to those of the untreated control. All measurements were performed in triplicate and repeated in three independent experiments.

### 2.5. Isobolographic Analysis of AKBA and Cis Interaction in A549 Cells

Isobolographic analysis was conducted to evaluate the interaction between AKBA and Cis in A549 cells. The half-maximal inhibitory concentration (IC50) values for AKBA (≥98% purity, Santa Cruz Biotechnology, USA) and Cis (≥99% purity, Thermo Fisher Scientific, USA), both individually and in combination, were determined from MTS assay data. For these experiments, A549 cells were seeded at a density of 5 × 10^3^ cells/well in 100 µL of complete medium in 96-well plates and treated with increasing concentrations of AKBA and Cis, either alone or in fixed-ratio combinations.

The combination index (CI) was calculated using the Chou–Talalay method [11], which quantitatively defines drug interactions. CI values were interpreted as follows: CI < 1 indicates synergism, CI = 1 an additive effect, and CI > 1 antagonism.

Graphical isobolograms were generated using CompuSyn software (ComboSyn Inc., Paramus, NJ, USA), which allows the visualization of drug interaction patterns based on dose–effect relationships. All experiments were conducted in triplicate unless otherwise specified.

### 2.6. Quantification of Cytokine Levels in AKBA- and Cis-Treated A549 Cells Using ELISA

Following treatment, A549 cells were seeded in 96-well plates at a density of 5 × 10^3^ cells/well in 100 µL of complete medium and incubated under standard conditions (37 °C, 5% CO_2_). After exposure to AKBA (≥98% purity, Santa Cruz Biotechnology, USA) and Cis (≥99% purity, Thermo Fisher Scientific, USA), cells were harvested and lysed in 50 µL of RIPA buffer or the lysis buffer provided by the ELISA kit (R&D Systems, Minneapolis, MN, USA) manufacturer. Lysates were centrifuged at 12,000× *g* for 10 min at 4 °C, and the resulting supernatants were collected for cytokine quantification.

The concentrations of interleukin-1β (IL-1β), interleukin-6 (IL-6), and tumor necrosis factor-alpha (TNF-α) were determined using commercially available sandwich ELISA kits (R&D Systems, Minneapolis, MN, USA), following the manufacturer’s protocols. Absorbance was measured at 450 nm using a Multiskan GO microplate reader (Thermo Fisher Scientific, USA). Cytokine levels were calculated based on standard curves generated for each cytokine. All experiments were conducted in triplicate unless otherwise specified.

### 2.7. Determination of Apoptosis in AKBA- and Cis-Treated A549 Cells Using Annexin V-FITC/PI Staining

Apoptosis was evaluated using an Annexin V–fluorescein isothiocyanate (FITC)/propidium iodide (PI) dual-staining apoptosis detection kit (BD Biosciences, San Jose, CA, USA). A549 cells were seeded at a density of 2 × 10^5^ cells/well in 6-well plates and incubated overnight in complete medium. Following treatment with AKBA (≥98% purity, Santa Cruz Biotechnology, USA), Cis (≥99% purity, Thermo Fisher Scientific, USA), or their combination, cells were harvested, washed twice with cold phosphate-buffered saline (PBS), and resuspended in 1× binding buffer at a final concentration of 1 × 10^6^ cells/mL.

Then, 100 µL of the cell suspension was incubated with 5 µL of Annexin V-FITC and 5 µL of PI for 15 min at room temperature in the dark. Following staining, 400 µL of binding buffer was added to each tube. The stained cells were immediately analyzed by flow cytometry (e.g., BD FACSCalibur or equivalent). Early apoptotic (Annexin V^+^/PI^−^) and late apoptotic (Annexin V^+^/PI^+^) cell populations were quantified and expressed as percentages relative to total cell counts. All experiments were conducted in triplicate unless otherwise specified.

### 2.8. Analysis of Gene Expression Levels of p53, Caspase-3, and NF-κB in AKBA- and Cis-Treated A549 Cells

The expression levels of apoptosis-related genes (p53 and caspase-3) and an inflammation-associated gene (NF-κB) were quantified using real-time quantitative polymerase chain reaction (RT-qPCR). Total RNA was extracted from A549 cells using TRIzol reagent (Invitrogen, Carlsbad, CA, USA) according to the manufacturer’s instructions. RNA purity and concentration were determined spectrophotometrically using a NanoDrop™ spectrophotometer (Thermo Fisher Scientific, USA), and RNA integrity was confirmed by agarose gel electrophoresis.

Complementary DNA (cDNA) synthesis was carried out using 1 µg of total RNA and the RevertAid First Strand cDNA Synthesis Kit (Thermo Scientific, Waltham, MA, USA) following the manufacturer’s instructions. RT-qPCR was performed using the SYBR™ Green PCR Master Mix (Applied Biosystems, Foster City, CA, USA) on the StepOnePlus™ Real-Time PCR System (Applied Biosystems). GAPDH was used as an internal control for normalization. Each reaction was run in triplicate.

The relative expression levels of the target genes were calculated using the 2^−ΔΔCt^ method. The primer sequences used for amplification are as follows:

- p53: F: 5′-CCTCTTCCTGCAGTACTCCC-3′, R: 5′-GCTCGCTTAGTGCTCCCT-3′
- Caspase-3: F: 5′-CTGGACTGTGGCATTGAGAC-3′, R: 5′-ACAAAGCGACTGGATGAACC-3′
- NF-κB: F: 5′-ATGTGGAGATCATTGAGCAGC-3′, R: 5′-CCTGGTCCTGTGTAGCCATT-3′
- GAPDH: F: 5′-GGAGCGAGATCCCTCCAAAAT-3′, R: 5′-GGCTGTTGTCATACTTCTCATGG-3′

### 2.9. Gene Ontology Enrichment Analysis of Differentially Expressed Genes in AKBA- and Cis-Treated A549 Cells

GO enrichment analysis was conducted to interpret the functional relevance of differentially expressed genes. GO term classification was based on the three main categories: biological process (BP), molecular function (MF), and cellular component (CC).

The analysis was performed using STRING database version 12.0 (https://string-db.org/, accessed on 15 July 2025), which integrates known and predicted protein–protein interactions. A *p*-value threshold of <0.05 was used to determine statistical significance, and multiple testing correction was applied using the false discovery rate method to minimize type I error.

GO terms with significant enrichment were further examined for functional clustering and to evaluate the potential biological roles and pathway involvements of the regulated genes.

### 2.10. Statistical Analysis

All data are presented as mean ± standard deviation (SD) from at least three independent experiments. Statistical analyses were performed using GraphPad Prism version 8.0 (GraphPad Software, San Diego, CA, USA). Comparisons among multiple groups were conducted using one-way analysis of variance (ANOVA), followed by Tukey’s post hoc test to identify significant pairwise differences. A *p*-value less than 0.05 (*p* < 0.05) was considered statistically significant.

### 2.11. Molecular Docking Analysis of AKBA with p53

AKBA was subjected to molecular docking analysis to predict its binding interactions with the tumor suppressor protein p53. The three-dimensional structure of human p53 was retrieved from the Protein Data Bank (PDB ID: 1TUPa https://www.rcsb.org/, accessed on 15 July 2025), and the structure of AKBA was obtained from the PubChem database (CID: 6918115, https://pubchem.ncbi.nlm.nih.gov/, accessed on 15 July 2025). Before docking, all water molecules and co-crystallized ligands were removed from the protein structure, and energy minimization was performed using the GROMOS96 force field via Swiss-PdbViewer (https://spdbv.unil.ch/, accessed on 15 July 2025).

Docking simulations were performed using AutoDock Vina (version 1.2.3). The grid box was centered to fully encompass the DNA-binding domain of p53, with dimensions adjusted to permit optimal ligand flexibility. The exhaustiveness parameter was set to 8 to ensure accurate sampling of binding conformations. Docking results were ranked based on binding affinity (kcal/mol).

The highest-scoring binding pose of AKBA was selected for detailed analysis. Interaction profiling revealed the presence of hydrogen bonding and hydrophobic contacts between AKBA and key residues in the DNA-binding groove of p53. All visualizations and interaction mappings were generated using Discovery Studio Visualizer 2021 (BIOVIA, Dassault Systèmes, San Diego, CA, USA). These findings suggest that AKBA may potentially modulate p53 signaling through direct interaction within its functional domain.

### 2.12. Molecular Dynamics Simulation of p53 in Complex with AKBA and Cis

To evaluate the structural stability and binding behavior of p53 in complex with AKBA and Cis, molecular dynamics simulations were conducted for 100 nanoseconds using GROMACS 2022 (version 2.5). Initial structures of the p53–AKBA and p53–Cis complexes were prepared with the CHARMM36 force field, and ligand topologies were generated via the CGenFF (CHARMM General Force Field, https://cgenff.com/, accessed on 15 July 2025) server.

Each complex was solvated in a cubic box filled with TIP3P water molecules and neutralized with Na^+^ and Cl^−^ ions to mimic physiological conditions. The systems underwent energy minimization followed by equilibration under constant volume (NVT) and constant pressure (NPT) ensembles at 300 K and 1 bar, respectively.

Production MD runs were performed for 100 ns. The resulting trajectories were analyzed using built-in GROMACS tools to compute the following parameters:

- Root Mean Square Deviation (RMSD) to assess structural deviation;
- Radius of Gyration (Rg) to evaluate compactness;
- Solvent Accessible Surface Area (SASA) to measure folding and exposure;
- Hydrogen bond count to estimate interaction strength;
- Root Mean Square Fluctuation (RMSF) per residue to examine local flexibility.

Data visualization and graphing were performed using GraphPad Prism (version 8.0, GraphPad Software, San Diego, CA, USA) and Seaborn libraries (version 0.11.2, Python library, Michael Waskom, USA). Comparative analysis revealed that both complexes were dynamically stable, with distinct interaction profiles and fluctuation behaviors.

### 2.13. Chemical Structure Illustration

The chemical structures of BA, AKBA, and Cis were retrieved from the PubChem database. Two-dimensional structures were generated using ChemDraw (version 20.0, PerkinElmer, Waltham, MA, USA) Professional (PerkinElmer, version 20.0, PerkinElmer, Waltham, MA, USA) and cross-verified with PubChem records to ensure structural accuracy. The structures are provided as Figure S1.

## 3. Results

### 3.1. Evaluation of Cytotoxic Effects of AKBA and Cis Using the MTS Assay

**Figure 1:**
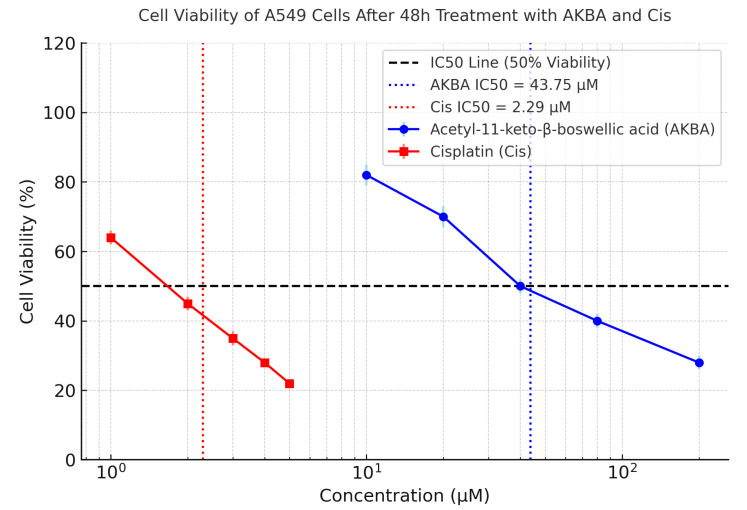
Dose-dependent cytotoxic effects of AKBA and Cis on A549 lung adenocarcinoma cells following 48 h treatment, as assessed using the MTS assay. Cell viability (%) is plotted against the logarithmic concentrations of each compound. AKBA reduced cell viability from approximately 82% at 10 µM to 28% at 200 µM, with an IC_50_ of 43.75 µM (blue dotted line). Cis reduced viability from approximately 64% at 1 µM to 22% at 5 µM, with an IC_50_ of 2.29 µM (red dotted line). The horizontal dashed black line indicates the 50% viability threshold. Data are presented as mean ± standard deviation (SD) from three independent experiments (*n* = 3). IC_50_ values were calculated by nonlinear regression using GraphPad Prism.

The results of the MTS assay demonstrated that both AKBA and Cis significantly inhibited the proliferation of A549 cells in a dose-dependent manner (*p* < 0.001). For AKBA, cell viability decreased from 82 ± 5.74% at 10 µM to 28 ± 1.96% at 200 µM, with a calculated IC_50_ value of 43.75 µM. In the case of Cis, cell viability declined from 64 ± 4.48% at 1 µM to 22 ± 1.54% at 5 µM, with an IC_50_ value of 2.29 µM (Figure 1).

One-way ANOVA revealed statistically significant differences across the concentration groups for both AKBA (F ≈ 139.6, *p* < 0.001) and Cis (F ≈ 198.4, *p* < 0.001). Post hoc analysis using Tukey’s HSD test confirmed that AKBA concentrations of 25 µM and above, and Cis concentrations of 1 µM and above, resulted in significantly lower viability compared to the control (vehicle-treated) group (*p* < 0.05).

### 3.2. Isobolographic Evaluation of the Combined Effects of AKBA and Cis

**Figure 2:**
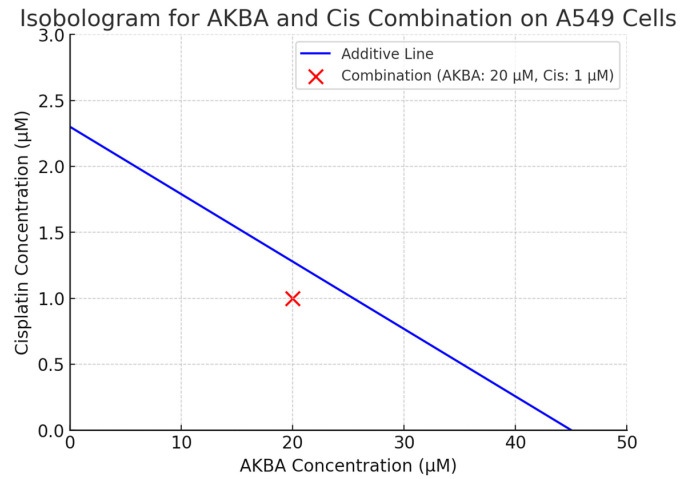
Isobologram showing the interaction between AKBA and Cis in A549 lung cancer cells, based on the Chou–Talalay method. The blue diagonal line represents the theoretical additive interaction derived from the individual IC_50_ values of AKBA (43.75 µM) and Cis (2.29 µM). The red dot denotes the tested combination (AKBA: 20 µM + Cis: 1 µM) that resulted in 50% inhibition of cell viability. As the red point falls below the additive line, the interaction is considered synergistic, with a calculated combination index (CI) of 0.894. Data are representative of three independent experiments.

Isobolographic analysis revealed that the combination of 20 µM AKBA and 1 µM Cis achieved 50% inhibition of cell viability, and the calculated combination index (CI = 0.894) indicated a synergistic effect. The area below the additive line in the isobologram represents the region of synergism (Figure 2).

The combination index (CI) was calculated using the Chou–Talalay method, as follows:

CI = D_1_/D_x1_ + D_2_/D_x2_

where

- D_1_: Concentration of AKBA in the combination (20 µM);
- Dx_1_: IC_50_ of AKBA alone (43.75 µM);
- D_2_: Concentration of Cis in the combination (1 µM);
- Dx_2_: IC_50_ of Cis alone (2.29 µM).

Since CI = 0.894 < 1, the combination of AKBA and Cis is classified as synergistic (Figure 2).

In addition to the representative combination of AKBA 20 µM + Cis 1 µM presented in Figure 2, multiple other dose combinations were also tested. The full viability results for all combinations are provided in Table S1. Among these, the 20 µM AKBA + 1 µM Cis combination was chosen for detailed presentation in the main text because it achieved ~50% inhibition of viability, corresponding to the IC_50_ point, and yielded the most representative synergistic outcome (CI = 0.894). Other combinations showed either additive or sub-additive interactions.

### 3.3. Assessment of Caspase-3/7 Activity in A549 Cells

**Figure 3:**
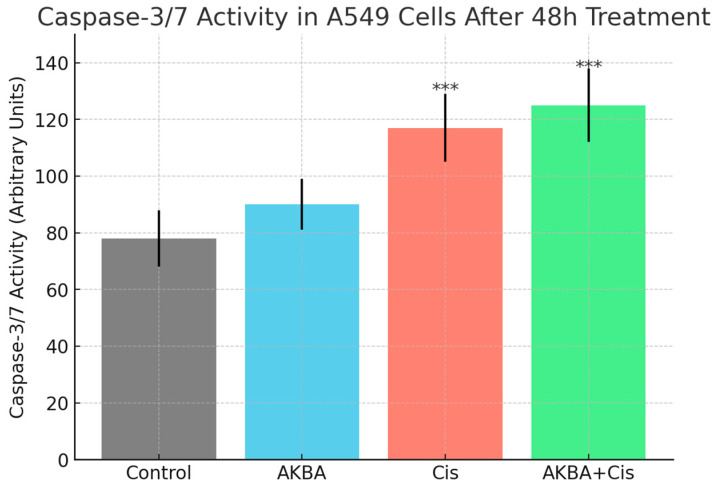
Caspase-3/7 activity in A549 lung cancer cells after 48 h treatment with AKBA, Cis, and their combination (AKBA + Cis). Caspase activity was measured using a luminescence-based assay and is expressed in arbitrary units. The AKBA + Cis combination group showed the highest caspase-3/7 activity, indicating enhanced apoptotic response. One-way ANOVA showed a significant difference among groups (*p* < 0.001). Tukey’s post hoc test revealed that both Cis and AKBA + Cis treatments significantly increased caspase activity compared to the control and AKBA groups (*** *p* < 0.001), while no significant difference was observed between Cis and AKBA + Cis (*p* = 0.62). Data are presented as mean ± SD (*n* = 3).

Caspase-3/7 activity analysis demonstrated that the highest enzymatic activity was observed in the AKBA + Cis combination group, whereas the lowest activity was detected in the control group. One-way ANOVA indicated a statistically significant difference among the groups (F ≈ 33.45, *p* < 0.001). According to the Tukey HSD post hoc test, both the Cis and AKBA + Cis treatment groups exhibited significantly elevated caspase-3/7 activity compared to the control and AKBA-only groups (*p* < 0.001). However, no significant difference was observed between the Cis and AKBA + Cis groups (*p* = 0.62) (Figure 3).

### 3.4. Quantification of Cytokine Levels in A549 Cells Treated with AKBA and Cis

Cytokine analysis demonstrated that the levels of IL-1β, IL-6, and TNF-α were significantly reduced in the combination therapy group compared to the control group, whereas reductions in some monotherapy groups did not reach statistical significance.

**Figure 4:**
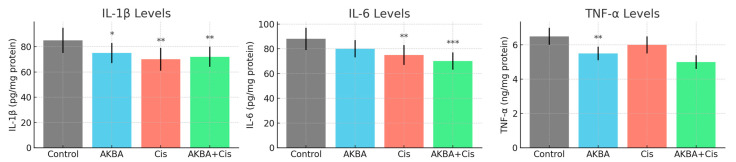
IL-1β, IL-6, and TNF-α levels in A549 lung cancer cells following 48 h treatments with AKBA, Cis, and their combination (AKBA + Cis). Cytokine concentrations are expressed as mean ± standard deviation (SD), *n* = 3. Statistical comparisons were performed using one-way ANOVA followed by Tukey’s post hoc test. In the left panel, IL-1β levels were significantly reduced in all treatment groups compared to the control (* *p* < 0.05 for AKBA, ** *p* < 0.01 for AKBA + Cis, *** *p* < 0.001 for Cis). The middle panel shows IL-6 levels, which were significantly decreased in the Cis (** *p* < 0.01) and AKBA + Cis (*** *p* < 0.001) groups, while AKBA alone had no significant effect (*p* = 0.08). In the right panel, TNF-α levels were significantly reduced in the AKBA and AKBA + Cis groups (** *p* < 0.01), whereas the Cis group did not show a significant change compared to the control.

- IL-1β levels were measured as 90.42 ± 4.24 pg/mg protein in the control group, 76.45 ± 8.12 in the AKBA group, 70.40 ± 4.32 in the Cis group, and 74.62 ± 5.58 in the AKBA + Cis group (ANOVA: F ≈ 11.36, *p* = 0.0012). Tukey’s HSD test confirmed that AKBA (*p* < 0.05), Cis (*p* < 0.001), and AKBA + Cis (*p* < 0.01) treatments significantly reduced IL-1β levels compared to the control.
- IL-6 levels were 85.12 ± 5.92 pg/mg protein in the control group, 72.20 ± 8.31 in the AKBA group, 66.40 ± 5.36 in the Cis group, and 62.62 ± 5.84 in the AKBA + Cis group (ANOVA: F ≈ 12.47, *p* = 0.0009). Tukey’s HSD test showed that Cis (*p* < 0.01) and AKBA + Cis (*p* < 0.001) treatments significantly decreased IL-6 levels compared to control, while the AKBA group showed no significant difference (*p* = 0.08).
- TNF-α levels were 6.24 ± 0.76 ng/mg protein in the control group, 4.64 ± 0.76 in the AKBA group, 5.84 ± 0.12 in the Cis group, and 4.82 ± 0.07 in the AKBA + Cis group (ANOVA: F ≈ 20.85, *p* = 0.0002). According to Tukey’s HSD test, both the AKBA (*p* < 0.01) and AKBA + Cis (*p* < 0.01) groups had significantly reduced TNF-α levels relative to the control, whereas the Cis group did not show a significant difference (*p* = 0.32) (Figure 4).

### 3.5. Assessment of Apoptotic Cell Death in A549 Cells Treated with AKBA and Cis

Annexin V-FITC/PI staining was used to quantify early and late apoptotic cell populations in A549 cells following treatment. The proportion of early apoptotic cells was 5.0 ± 1.2% in the control group, 18.0 ± 2.5% in the AKBA group, 23.0 ± 2.8% in the Cis group, and 34.0 ± 3.0% in the AKBA + Cis combination group. Correspondingly, late apoptotic cell percentages were 3.0 ± 0.8% in the control, 20.0 ± 2.6% in AKBA, 26.0 ± 2.9% in Cis, and 35.0 ± 3.2% in the AKBA + Cis group (mean ± SD, *n* = 3).

**Figure 5:**
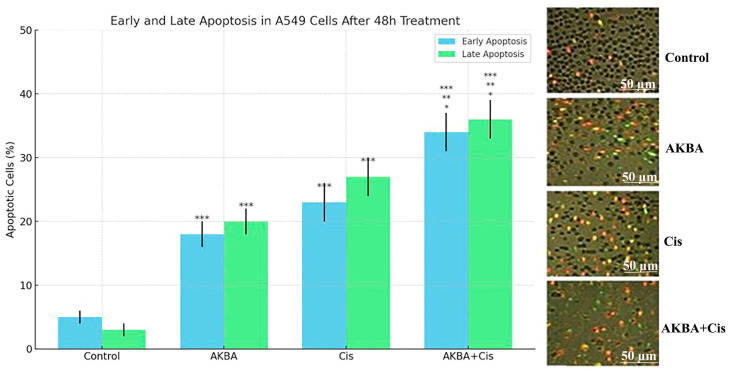
Quantitative and visual assessment of early and late apoptosis in A549 lung cancer cells after 48 h treatment with AKBA, Cis, and their combination (AKBA + Cis). The bar graph displays the percentages of early apoptotic (blue bars) and late apoptotic (green bars) cells, expressed as mean ± standard deviation (SD), based on three independent experiments (*n* = 3). Statistical differences among groups were evaluated using one-way ANOVA (early apoptosis: F ≈ 66.82, *p* < 0.0001; late apoptosis: F ≈ 92.67, *p* < 0.0001), followed by Tukey’s post hoc test. Significant differences are indicated as follows: *** *p* < 0.001 vs. control; ** *p* < 0.01 vs. AKBA; * *p* < 0.05 vs. Cis. All treatment groups showed significantly higher apoptosis compared to the control group (*** *p* < 0.001). Additionally, the combination group (AKBA + Cis) exhibited significantly greater early (*p* = 0.006) and late (*p* < 0.001) apoptosis compared to AKBA (** *p* < 0.01), as well as significantly greater early (*p* = 0.03) and late (*p* = 0.02) apoptosis compared to Cis (* *p* < 0.05). Representative fluorescence microscopy images of Annexin V-FITC/PI-stained cells from each group (Control, AKBA, Cis, AKBA + Cis) are shown on the right, with green fluorescence indicating early apoptotic cells and red fluorescence indicating late apoptotic or necrotic cells. The AKBA + Cis treatment resulted in the most pronounced apoptotic activity among all groups. The AKBA + Cis treatment resulted in the most pronounced apoptotic activity among all groups. Scale bars: 50 µM.

One-way ANOVA analysis revealed statistically significant differences among the groups for both early apoptosis (F ≈ 66.82, *p* < 0.0001) and late apoptosis (F ≈ 92.67, *p* < 0.0001). Tukey’s post hoc test demonstrated that all treatment groups (AKBA, Cis, and AKBA + Cis) exhibited significantly elevated early and late apoptosis compared to the control group (*** *p* < 0.001). Furthermore, the combination treatment (AKBA + Cis) resulted in significantly higher early (*p* = 0.006) and late (*p* < 0.001) apoptosis compared to AKBA alone, and significantly higher early (*p* = 0.03) and late (*p* = 0.02) apoptosis compared to Cis alone. No statistically significant difference was observed between AKBA and Cis groups in terms of early (*p* = 0.27) or late (*p* = 0.10) apoptotic rates (Figure 5).

**Figure 6:**
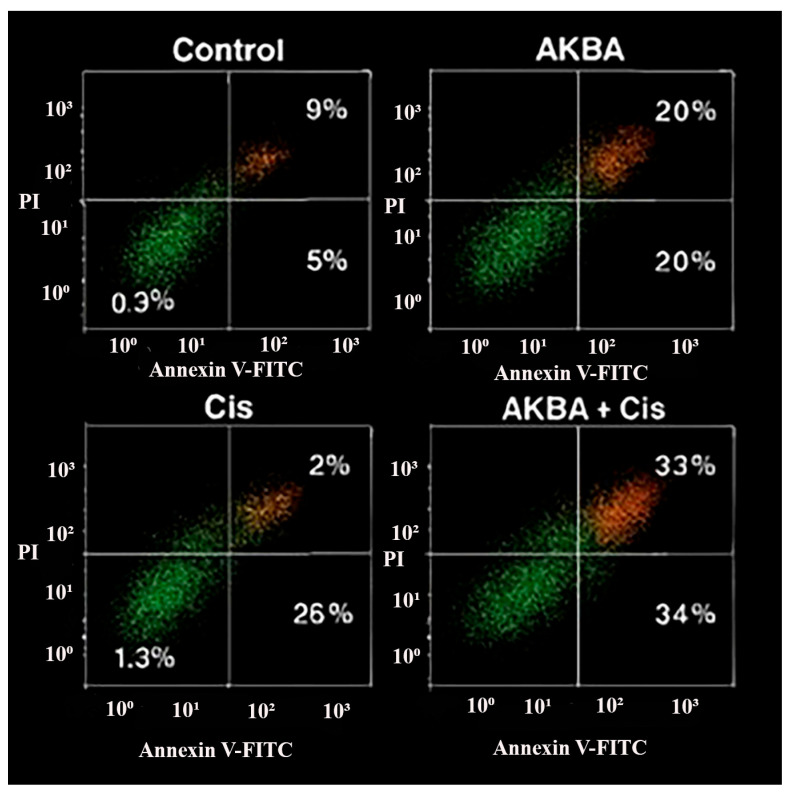
Representative Annexin V-FITC/PI flow cytometry dot plots of A549 lung cancer cells after 48 h of treatment with AKBA, Cis, and their combination (AKBA + Cis). The quadrants indicate viable cells (Annexin V^−^/PI^−^, lower left), early apoptotic cells (Annexin V^+^/PI^−^, lower right), late apoptotic cells (Annexin V^+^/PI^+^, upper right), and necrotic cells (Annexin V^−^/PI^+^, upper left). Percentages represent the proportion of cells in each quadrant. The combination group (AKBA + Cis) demonstrated the highest levels of both early and late apoptosis compared with single-agent treatments and the control group.

Flow cytometric analysis using Annexin V-FITC/PI staining demonstrated a significant increase in apoptotic cell populations following treatment with AKBA, Cis, and their combination compared with the control group. As shown in Figure 6, the proportion of early apoptotic cells (Annexin V^+^/PI^−^, lower right quadrant) rose from 5% in the control group to 20% in the AKBA group, 26% in the Cis group, and 34% in the AKBA + Cis group. Similarly, late apoptotic cells (Annexin V^+^/PI^+^, upper right quadrant) increased from 9% in the control to 20% with AKBA, 23% with Cis, and 33% with the combination treatment. The AKBA + Cis group exhibited the highest rates of both early and late apoptosis, confirming a synergistic pro-apoptotic effect in A549 cells.

### 3.6. Assessment of Apoptosis- and Inflammation-Related Gene Expression in A549 Cells Treated with AKBA and Cis

RT-qPCR analysis demonstrated that the combination treatment (AKBA + Cis) significantly upregulated p53 (~3.2-fold) and caspase-3 (~3.6-fold) gene expression, while downregulating NF-κB expression by approximately 54% (~0.46-fold) compared to the control group. One-way ANOVA revealed statistically significant differences among the groups for all three genes. Compared to the control, the AKBA + Cis combination induced a highly significant increase in p53 (*p* < 0.001) and caspase-3 (*p* < 0.001), and a significant decrease in NF-κB expression (*p* < 0.001).

**Figure 7:**
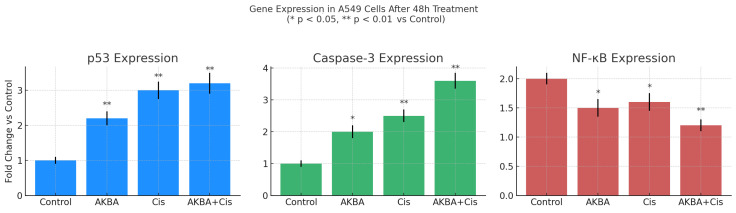
Relative expression levels of apoptosis- and inflammation-related genes in A549 cells following 48 h treatment with AKBA, Cis, and their combination (AKBA + Cis). RT-qPCR analysis was conducted to assess the mRNA expression of p53, caspase-3, and NF-κB in A549 cells treated with AKBA, Cis, or the combination of both agents. Gene expression levels were normalized to a housekeeping gene and calculated as fold changes relative to the untreated control group using the 2^−ΔΔCt^ method. The combination treatment (AKBA + Cis) resulted in a significant upregulation of p53 (~3.2-fold) and caspase-3 (~3.6-fold), as well as a notable downregulation of NF-κB (~0.46-fold), compared to the control. Monotherapies with AKBA and Cis also caused statistically significant but less pronounced effects on all three genes. Data are presented as mean ± standard deviation (SD) from three independent experiments (*n* = 3). Statistical analysis was performed using one-way ANOVA followed by Tukey’s post hoc test. Statistical significance levels compared to the control group are denoted as follows: *p* < 0.05 (*), *p* < 0.01 (**).

AKBA and Cis monotherapies also caused significant alterations in gene expression: p53 (AKBA: *p* = 0.003; Cis: *p* < 0.001), caspase-3 (AKBA: *p* = 0.02; Cis: *p* = 0.006), and NF-κB (AKBA: *p* = 0.004; Cis: *p* = 0.03) levels were all significantly modulated compared to the control. The combination treatment demonstrated a synergistic effect, enhancing apoptotic gene expression and suppressing inflammatory gene expression more effectively than either agent alone, even at lower doses (Figure 7).

### 3.7. Gene Ontology Analysis of Apoptotic and Inflammatory Pathways

GO enrichment analysis conducted for the p53 and caspase-3 genes revealed their involvement in key biological processes associated with apoptosis. For p53, significantly enriched biological processes included “regulation of apoptotic process,” “cell cycle arrest,” and “DNA damage response,” underscoring its role as a central regulator initiating apoptosis under cellular stress and DNA damage conditions.

For Caspase-3, the most significant enrichment was observed in GO terms related to its effector functions, such as “execution phase of apoptosis” and “cysteine-type endopeptidase activity.” Additionally, caspase-3 was found to be predominantly localized in cellular components including the “cytosol” and “apoptotic body.”

**Figure 8:**
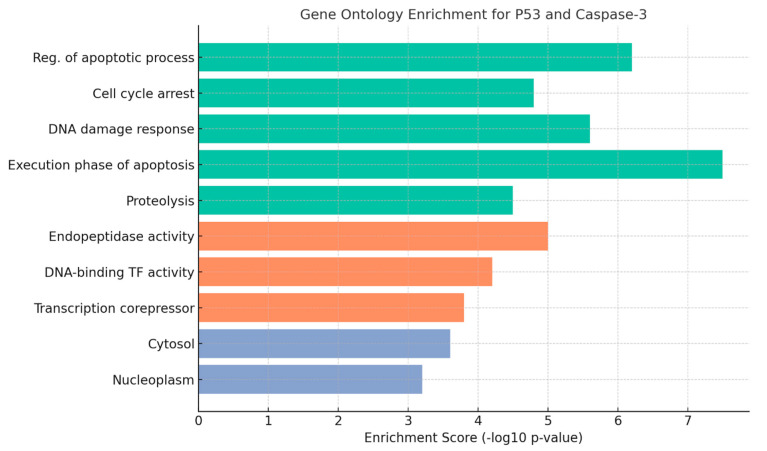
GO enrichment analysis for p53 and caspase-3 genes. This figure displays the top GO terms grouped into three categories: Biological Processes (BP, green bars), Molecular Functions (MF, orange bars), and Cellular Components (CC, blue bars). Biological processes such as “regulation of apoptotic process,” “cell cycle arrest,” and “DNA damage response” highlight the central roles of p53 and caspase-3 in apoptosis and cellular stress regulation. Molecular function enrichment includes “endonuclease activity” and “DNA-binding transcription factor activity,” emphasizing their mechanistic contributions. Cellular component terms indicate primary localization in “cytosol” and “nucleoplasm.” Enrichment scores are represented as −log10 (*p*-value), with higher values indicating stronger statistical significance of functional association.

Evaluation of enrichment scores based on −log10 (*p*-value) further substantiated the pivotal roles of both p53 and caspase-3 within the apoptosis pathway. These findings indicate that both genes are critically involved in treatment-induced apoptosis and are functionally enriched in processes that govern programmed cell death (Figure 8).

### 3.8. Molecular Docking Analysis of AKBA with p53 Protein

Molecular docking simulations were performed to predict the binding interaction of AKBA with the DNA-binding domain of the p53 tumor suppressor protein. The 3D structure of p53 (PDB ID: 2OCJ) and AKBA (PubChem CID: 12358603) were prepared for docking using AutoDock Vina. Water molecules and heteroatoms were removed, and energy minimization was conducted using the GROMOS96 force field.

**Figure 9:**
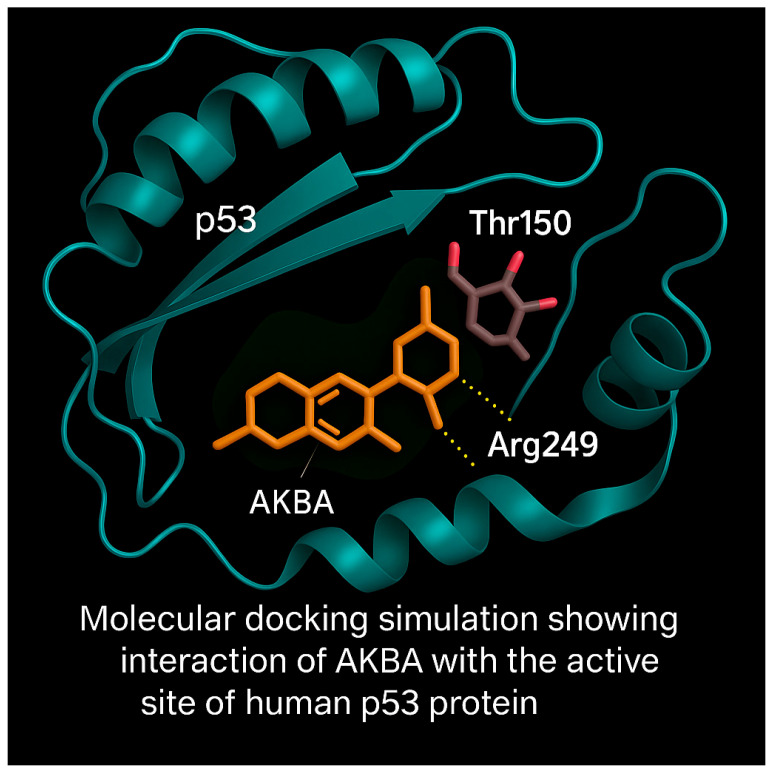
Molecular docking analysis illustrating the interaction between AKBA and the DNA-binding domain of the human p53 protein. AKBA (orange) is shown bound within the p53 binding pocket, forming hydrogen bonds (yellow dashed lines) with Arg249 and Thr150. The p53 backbone is visualized as a ribbon diagram, highlighting the structural context of the binding site.

The docking analysis revealed that AKBA binds within the active site cavity of p53 with a binding affinity of −8.1 kcal/mol. Visualization of docking poses showed hydrogen bond interactions with Arg249 and proximity to Thr150, both critical residues within the DNA-binding domain (Figure 9).

These interactions suggest that AKBA may stabilize or modulate p53 activity, potentially enhancing its tumor suppressor functions. The docking model highlights AKBA’s potential as a targeted modulator of p53-mediated apoptosis.

### 3.9. Molecular Dynamics Simulation of p53–AKBA and p53–Cis Complexes

To evaluate the dynamic behavior and stability of the p53–AKBA and p53–Cis complexes, a 100 ns molecular dynamics simulation was conducted.

**Figure 10:**
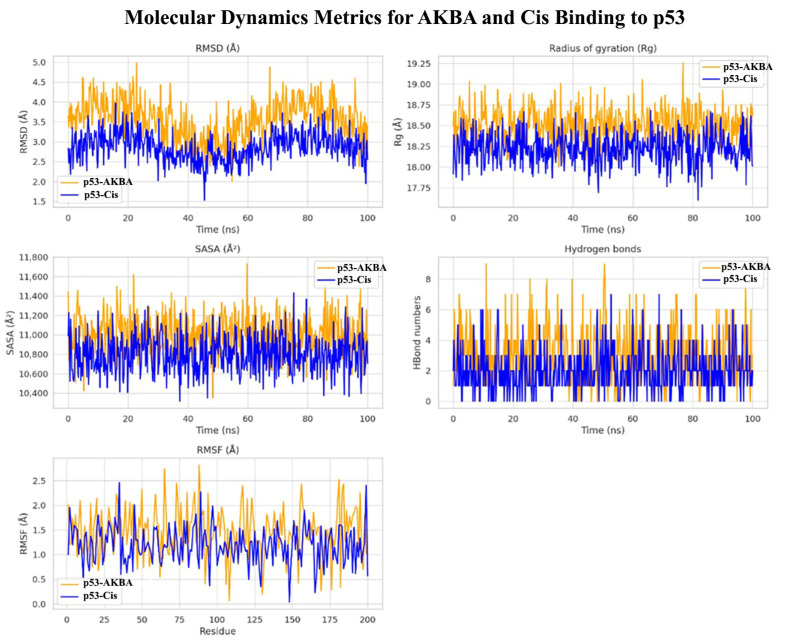
Molecular dynamics simulation metrics comparing p53–AKBA (orange) and p53–Cis (blue) complexes over 100 ns. Top row: RMSD (Root Mean Square Deviation, in Å) and Rg (Radius of Gyration, in Å). Middle row: SASA (Solvent Accessible Surface Area, in Å^2^) and hydrogen bond counts. Bottom row: RMSF (Root Mean Square Fluctuation, in Å) per residue. The p53–AKBA complex formed more hydrogen bonds and showed distinct flexibility profiles in terminal domains. Å (Angström) is a unit of length equal to 0.1 nanometers (nm), commonly used to represent atomic-scale distances in molecular dynamics.

RMSD plots revealed that both complexes remained stable throughout the simulation, with p53–Cis showing slightly lower fluctuations (average RMSD ~2.6 Å) compared to p53–AKBA (~3.1 Å), indicating greater structural rigidity (Figure 10, top left). Rg analysis demonstrated comparable compactness in both complexes (18.4–18.9 Å range), with p53–Cis maintaining slightly more compact structure (top right).

SASA profiles showed marginally lower solvent exposure in the p53–Cis complex, suggesting tighter folding (middle left). Notably, the p53–AKBA complex exhibited a higher number of hydrogen bonds throughout the simulation (middle right), implying greater stabilization via non-covalent interactions.

RMSF plots highlighted higher residue flexibility in the loop and terminal regions of p53 in the AKBA complex (bottom left), which may indicate allosteric modulation potential. Collectively, the data suggest that while p53–Cis maintains tighter folding, p53–AKBA forms more extensive and potentially functional interactions.

## 4. Discussion

Lung cancer remains one of the most prevalent and deadly malignancies worldwide. Despite advances in chemotherapeutic strategies, systemic toxicity and acquired resistance continue to limit therapeutic efficacy. Therefore, combination therapies that enhance tumoricidal activity while minimizing toxicity are of paramount interest. In this context, our study demonstrates that co-treatment with AKBA, a natural pentacyclic triterpenoid, and Cis, a standard chemotherapeutic agent, exerts a synergistic anti-tumor effect on A549 human lung cancer cells. This synergism was confirmed by isobolographic analysis (CI = 0.894) and was further supported by a comprehensive set of cellular and molecular data, including viability assays, apoptosis markers, gene expression profiling, cytokine quantification, and GO enrichment analysis. Among the different dose combinations tested, AKBA 20 µM + Cis 1 µM was selected as the representative regimen because it produced ~50% inhibition of cell viability, corresponding to the IC_50_ point, and demonstrated the clearest synergistic interaction (CI = 0.894). Importantly, this combination achieved significant efficacy at relatively low concentrations of both agents, supporting a dose-sparing strategy that may reduce systemic toxicity. Other tested combinations produced additive or less pronounced effects, which further justified the selection of this dose pair for detailed analysis.

MTS analysis showed that AKBA and Cis individually suppressed A549 cell proliferation in a dose-dependent manner, while the combined treatment achieved equivalent cytotoxicity at significantly lower doses. Mechanistically, AKBA’s multidimensional properties—including antioxidant and anti-inflammatory actions—appear to counterbalance the oxidative and inflammatory burden associated with Cis-induced DNA damage [7,12]. This dual targeting of intracellular stress and inflammation allows the combination to more effectively trigger apoptotic pathways.

Notably, suppression of NF-κB signaling emerged as a critical component of this synergy. NF-κB is a key mediator of inflammation and survival signaling in cancer cells and contributes to the development of chemoresistance [13]. Inhibiting NF-κB not only downregulates inflammatory cytokines (IL-1β, IL-6, TNF-α) but also enhances the expression of pro-apoptotic genes such as p53 and caspase-3, thereby facilitating apoptotic progression. Flow cytometry analysis using Annexin V/PI and caspase-3/7 enzymatic assays demonstrated that co-treatment with AKBA and Cis significantly increased both early and late apoptosis, along with elevated caspase activity. Caspase-3, a central executioner of apoptosis, initiates the systematic dismantling of cellular structures, and its activation is a definitive hallmark of programmed cell death [14].

RT-qPCR results supported these observations by showing substantial upregulation of p53 and caspase-3, accompanied by marked downregulation of NF-κB. Given that p53 plays a central role in sensing DNA damage and regulating apoptosis, and that Cis is known to activate the p53 pathway through DNA cross-linking, the data suggest that AKBA potentiates this effect by relieving NF-κB–mediated anti-apoptotic pressure. The observed synergism, therefore, reflects not only enhanced pro-apoptotic signaling but also suppression of inflammatory and survival pathways [15,16].

GO enrichment analysis further contextualized these findings by identifying significant associations of p53 with biological processes such as “regulation of apoptotic process,” “cell cycle arrest,” and “DNA damage response,” and of caspase-3 with “execution phase of apoptosis” and “endopeptidase activity.” These results affirm the central roles of these genes in apoptosis and reinforce the biological plausibility of the molecular mechanisms underlying the observed phenotypes [17].

Moreover, cytokine profiling revealed significant reductions in IL-1β, IL-6, and TNF-α levels in the combination group, supporting an anti-inflammatory and immunomodulatory role. Chronic inflammation is a well-established driver of tumor progression and chemoresistance [18]. By mitigating pro-inflammatory signaling, particularly through NF-κB inhibition, the AKBA + Cis regimen may improve the therapeutic index of chemotherapy and reshape the tumor microenvironment to favor apoptosis over survival [19,20]. The biological relevance of these cytokine changes is considerable. IL-6 and TNF-α are central activators of NF-κB, driving angiogenesis, epithelial–mesenchymal transition, and resistance to apoptosis, while IL-1β has been implicated in promoting immunosuppression and metastatic spread within the tumor microenvironment. Their downregulation may therefore attenuate tumor-promoting inflammation, reduce survival signaling, and restore the balance toward pro-apoptotic and immune-mediated tumor clearance. Such immunomodulatory effects suggest that the AKBA + Cis combination not only acts directly on tumor cells but may also influence the broader tumor microenvironment by diminishing inflammatory cues that sustain malignant progression. The potential of AKBA derivatives to synergize with chemotherapeutic agents is also supported by previous reports showing that AKBA, another Boswellia derivative, induces G0/G1 cell cycle arrest and enhances Cis-induced apoptosis in various cancer models [21,22]. In addition to its anti-tumor effects, AKBA may also mitigate systemic toxicity during chemotherapy through its antioxidant and anti-inflammatory properties [23].

Our study demonstrates that AKBA, when combined with Cis, significantly enhances apoptosis in A549 NSCLC cells, primarily through upregulation of pro-apoptotic genes p53 and caspase-3 and downregulation of the inflammatory transcription factor NF-κB. These findings align with and expand upon previous studies reporting the anti-cancer and chemosensitizing effects of AKBA derivatives in various tumor models.

Multiple studies have emphasized the role of AKBA, a potent derivative of AKBA, in enhancing the efficacy of Cis by inducing apoptosis and cell cycle arrest. Lv et al. demonstrated that AKBA enhances Cis sensitivity in NSCLC cells through p21-dependent cell cycle arrest, increased apoptosis, and autophagy suppression [5]. Our results support this by confirming enhanced apoptosis and p53 activation with AKBA + Cis combination therapy, although we did not directly assess autophagy-related pathways. Similarly, Al-Bahlani et al. showed that AKBA potentiated Cis-induced apoptosis in gastric cancer cells via a p53-mediated mechanism, with concurrent suppression of Akt and NF-κB signaling [24], consistent with our observed NF-κB downregulation and p53 upregulation. Moreover, the role of AKBAs in suppressing inflammatory pathways—especially NF-κB—has been well-documented. Trivedi et al. and Guo et al. highlighted NF-κB’s multifaceted involvement in inflammation, apoptosis resistance, and tumor progression, and emphasized the therapeutic potential of NF-κB inhibition in cancer therapy [12,25]. Our results reinforce this, demonstrating that AKBA + Cis co-treatment effectively attenuates NF-κB expression, which likely contributes to the observed apoptotic enhancement.

Schmiech et al. further validated the pro-apoptotic effects of 11-keto-α-boswellic acid in triple-negative breast cancer, showing its ability to induce apoptosis both in vitro and in xenograft models [26]. Although our study focused solely on in vitro A549 cells, the increased apoptotic index and Annexin V positivity in the AKBA + Cis group suggest translational potential, pending in vivo validation. GO enrichment analysis in our study provided further insight, revealing that p53 was associated with key apoptotic biological processes such as “DNA damage response” and “cell cycle arrest,” while caspase-3 was linked to the “execution phase of apoptosis” and “cysteine-type endopeptidase activity.” These findings are in line with previous mechanistic studies [5,27,28], suggesting conserved apoptotic pathways are modulated by AKBA treatment.

Additionally, our results complement the findings of Ragab et al., who described the molecular mechanisms underlying AKBA-induced cytotoxicity, including PARP cleavage, Bax upregulation, and caspase activation [29]. These pathways are likely activated in our AKBA + Cis treatment group, as evidenced by caspase-3 gene expression upregulation and increased early and late apoptotic cell populations. While the clinical use of boswellic acid derivatives remains under investigation, previous human trials using boswellic acid-based formulations, such as topical applications to mitigate radiotherapy-induced dermatitis, have reported excellent tolerability [30]. This supports the potential translational safety of boswellic acid-based therapies. Nonetheless, the in vitro nature of our study poses limitations in terms of pharmacokinetics and systemic effects.

Furthermore, molecular docking and molecular dynamics simulations provided deeper mechanistic insight into the interaction between AKBA and p53. Docking studies performed with AutoDock Vina, a fast and accurate open source docking engine [31], revealed stable hydrogen bonding between AKBA and critical residues such as Arg249 and Thr150 within the DNA binding domain of p53. Molecular dynamics simulations confirmed the dynamic stability of p53 AKBA and p53 Cis complexes over 100 ns, showing sustained RMSD plateaus, consistent Rg values, stable SASA profiles, persistent hydrogen bonds, and reduced terminal flexibility as captured by RMSF analysis. These characteristics are recognized as indicative of structurally robust protein ligand interactions [32]. These in silico findings reinforce the experimental data by supporting the hypothesis that AKBA may directly stabilize p53 and thereby enhance apoptotic signaling. The biological relevance of the observed AKBA–p53 interactions lies in the central role of p53 as a transcription factor that regulates cell fate following DNA damage. Stabilization of the DNA-binding domain of p53 by AKBA, as indicated by our docking and dynamics simulations, may enhance its ability to transactivate downstream pro-apoptotic targets such as BAX, PUMA, and caspase-3. This would not only lower the apoptotic threshold of A549 cells but also potentiate the cytotoxic activity of cisplatin, which induces apoptosis primarily through DNA cross-linking and p53 activation. Thus, the in silico evidence of stable AKBA–p53 binding provides a mechanistic explanation for the enhanced apoptosis observed experimentally, linking structural interactions with functional apoptotic outcomes.

Future research should validate these findings in vivo using both xenograft and orthotopic NSCLC models to better mimic tumor heterogeneity, stromal interactions, and drug pharmacokinetics. Comprehensive pharmacokinetic and pharmacodynamic studies are warranted to determine optimal dosing, bioavailability, and tissue distribution of AKBA in combination with Cis. Mechanistic experiments employing gene silencing or CRISPR-mediated knockout of p53, NF-κB, and caspase-3, as well as pharmacologic inhibition assays, will be critical to confirm causal roles of these pathways. Furthermore, mitochondrial apoptotic events, including alterations in membrane potential, cytochrome c release, and caspase-9 activation, should be investigated to establish the involvement of intrinsic apoptosis. Ultimately, if these preclinical findings are corroborated, early-phase clinical studies could evaluate AKBA as a dose-sparing chemosensitizer in combination with Cis, aiming to enhance therapeutic efficacy while reducing systemic toxicity in NSCLC and potentially other solid tumors.

A major strength of this study lies in its multi-level experimental design, integrating cytotoxicity assays, apoptosis quantification, gene expression profiling, cytokine analysis, GO enrichment, and in silico molecular modeling. Specifically, molecular docking and molecular dynamics simulations provided critical structural insights into the interaction of AKBA, a bioactive derivative of boswellic acid, with the DNA binding domain of p53. These computational findings revealed stable hydrogen bonding and dynamic stability of the p53 and AKBA complex, supporting and complementing the experimental data and reinforcing the proposed mechanism of p53 mediated apoptosis enhancement.

Furthermore, the use of low-dose combinations to achieve high apoptotic efficacy highlights the therapeutic potential of dose-minimization strategies in reducing chemotherapy-associated toxicity. However, several limitations should be acknowledged. First, all experiments were conducted exclusively under in vitro conditions using a single NSCLC cell line (A549), which may not fully recapitulate the complexity of tumor heterogeneity, cell–cell interactions, or drug pharmacokinetics observed in vivo. Second, the study did not incorporate functional knockdown or inhibition assays (e.g., siRNA, CRISPR, or pharmacologic inhibitors) to validate the mechanistic roles of key targets such as p53 and caspase-3. Such analyses would further strengthen the causal interpretation of their involvement in the observed apoptotic response. Third, although RT-qPCR confirmed transcriptional changes in p53, caspase-3, and NF-κB, these results were not validated at the protein level. The absence of complementary protein-level assays (e.g., Western blotting or ELISA) limits the confirmation of whether mRNA upregulation translated into functional protein expression. Fourth, cell viability and proliferation assays were assessed only at a single 48 h time point; earlier measurements (e.g., 24 h) would provide valuable insights into the short-term cytotoxic effects of AKBA and Cis, and future studies should incorporate multiple time points to better characterize the temporal dynamics of apoptosis and proliferation inhibition. Finally, network pharmacology analysis should be integrated in future studies to evaluate whether the observed effects of AKBA and Cis align with broader molecular interaction networks and previously reported datasets, thereby enhancing the translational relevance of our findings.

To translate these findings into clinical relevance, future studies should aim to validate the therapeutic efficacy of the AKBA and Cis combination in appropriate in vivo models. Comprehensive pharmacokinetic and pharmacodynamic analyses are warranted to determine optimal dosing strategies, bioavailability, and tissue distribution. In addition, functional studies involving gene silencing or CRISPR mediated knockout of p53, NFκB, or caspase 3 would help elucidate the specific molecular contributions to the observed synergistic effects. Investigating key mitochondrial events such as changes in membrane potential, cytochrome c release, and the activation of upstream initiator caspases including caspase 9 could further clarify the involvement of intrinsic apoptotic pathways. If corroborated in preclinical animal models and clinical settings, the AKBA plus Cis combination may represent a promising therapeutic strategy for lung cancer, offering enhanced efficacy with potentially reduced systemic toxicity.

## 5. Conclusions

This study demonstrates that the combination of AKBA and Cis exerts a synergistic pro-apoptotic effect on A549 non-small-cell lung cancer cells. The enhanced cytotoxicity was associated with the upregulation of p53 and caspase-3 and the suppression of NF-κB, a key regulator of inflammation and cell survival. Gene ontology enrichment analysis further supported the involvement of biological processes related to apoptosis, DNA damage response, and cell cycle arrest.

In silico docking and molecular dynamics simulations revealed that AKBA, the most biologically active derivative of boswellic acid, forms a stable interaction with the DNA-binding domain of p53, suggesting a potential role in modulating its activity.

These findings suggest that AKBA and Cis may represent a promising combination strategy to enhance apoptotic signaling and counteract resistance mechanisms in lung cancer. However, further in vivo and mechanistic studies are warranted to validate these effects and determine their clinical applicability.

## Data Availability

The human NSCLC cell line A549 (ATCC^®^ CCL-185™) was obtained from the American Type Culture Collection (ATCC, Manassas, VA, USA). All details about the study can be obtained from the corresponding author.

## Supplementary Materials

The following supporting information can be downloaded at: https://www.mdpi.com/article/10.3390/cimb47090785/s1.
