# Supplementary material for: Synergistic Induction of Apoptosis by Boswellic Acid and Cisplatin in A549 Lung Cancer Cells Through NF-κB Modulation and p53 Pathway Activation
Source: Curr Issues Mol Biol. 2025 Sep 22;47(9):785. doi: 10.3390/cimb47090785 (PMC12468240; doi:10.3390/cimb47090785)
Supplement: Supplementary file 1 [file cimb-47-00785-s001.zip › Supplementary Table S1.pdf]

Supplementary Table S1. Cell Viability (%) of A549 Cells Treated with AKBA and Cis Alone or in Combination for 48 h (MTS Assay, Mean ± SD, n = 3)

AKBA (μM)	Cis (μM)	Cell Viability (%)	Combination Index (CI)	Interaction Type
0	0	100 ± 2.1	N/A	N/A
10	0	82 ± 5.7	N/A	N/A
25	0	65 ± 4.8	N/A	N/A
50	0	48 ± 3.9	N/A	N/A
100	0	39 ± 2.5	N/A	N/A
200	0	28 ± 2.0	N/A	N/A
0	1	64 ± 4.5	N/A	N/A
0	2	40 ± 3.2	N/A	N/A
0	3	32 ± 2.8	N/A	N/A
0	4	25 ± 2.4	N/A	N/A
0	5	22 ± 1.5	N/A	N/A
20	1	50 ± 2.8	0.894	Synergism

Footnote: CI (Combination Index) and interaction type are reported only for the representative combination treatment (AKBA 20 μM + Cis 1 μM), which demonstrated synergism (CI = 0.894). For single-agent treatments, CI is not applicable (N/A).
