# Supplementary figures and images for: Synergistic Induction of Apoptosis by Boswellic Acid and Cisplatin in A549 Lung Cancer Cells Through NF-κB Modulation and p53 Pathway Activation
Source: Curr Issues Mol Biol. 2025 Sep 22;47(9):785. doi: 10.3390/cimb47090785 (PMC12468240; doi:10.3390/cimb47090785)

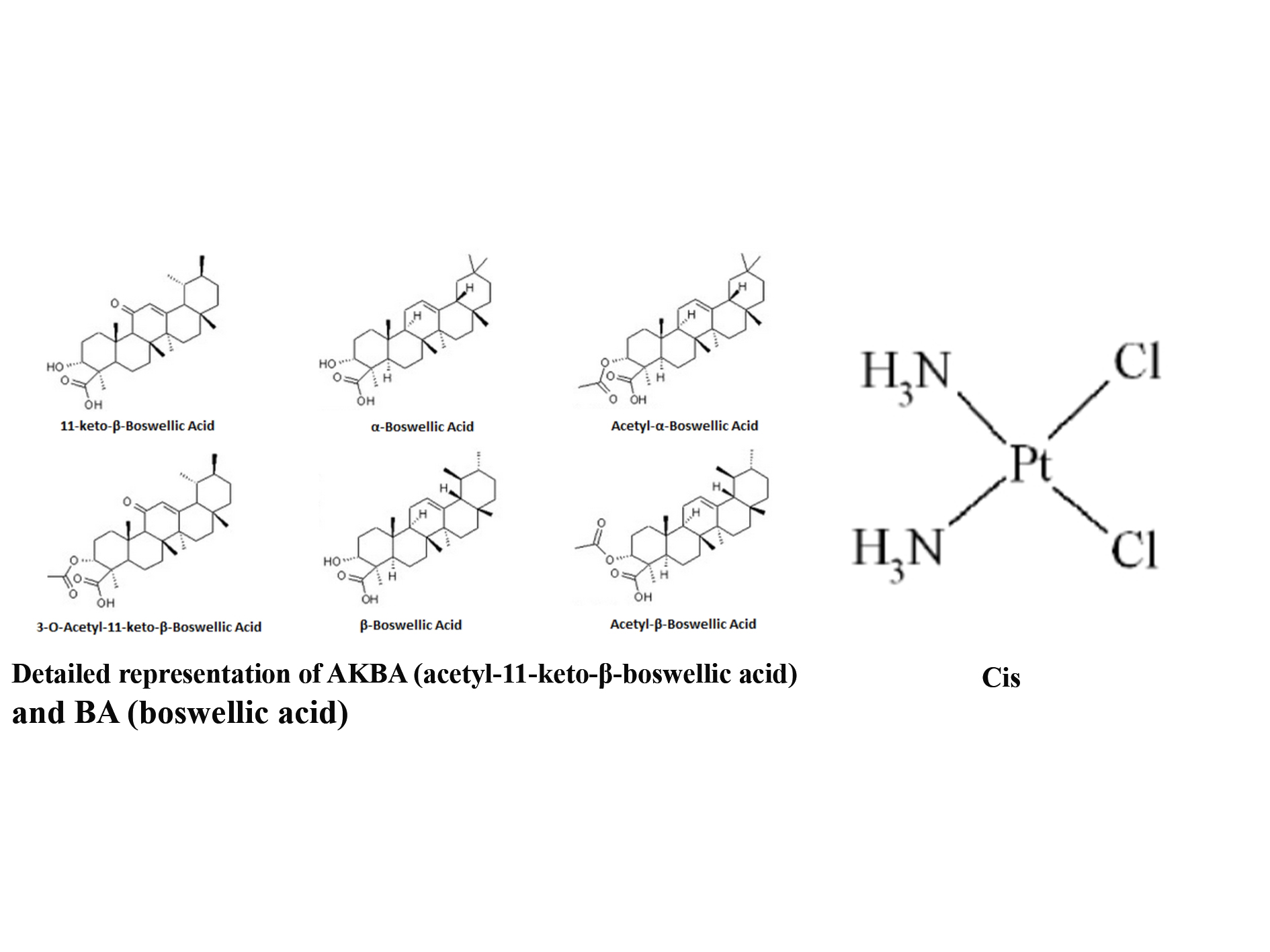

Supplement: Supplementary file 1 [file cimb-47-00785-s001.zip › Supplementary Figure S1.jpg]
